# Investigation on Crystal-Structure, Thermal and Electrical Properties of PVDF Nanocomposites with Cobalt Oxide and Functionalized Multi-Wall-Carbon-Nanotubes

**DOI:** 10.3390/nano12162796

**Published:** 2022-08-15

**Authors:** Farhan, Shabir Ahmad, Hameed Ullah, Zia Ur Rehman, Mohsan Nawaz, Imad Uddin, Anand Parkash, Hatem R Alamri, Norah Salem Alsaiari, Muhammad Sufyan Javed

**Affiliations:** 1 Department of Chemistry, Islamia College University, Peshawar 25120, Pakistan; 2 Department of Chemistry, Hazara University, Mansehra 21120, Pakistan; 3 Department of Chemistry, University of Haripur, Haripur 22620, Pakistan; 4 School of Chemistry and Chemical Engineering, Shaanxi Normal University, Chang’an West Street 620, Xi’an 710119, China; 5 Physics Department, Aljamoum University College, Umm Al-Qura University, Makkah 25375, Saudi Arabia; 6 Department of Chemistry, College of Science, Princess Nourah bint Abdulrahman University, P.O. Box 84428, Riyadh 11671, Saudi Arabia; 7 School of Physical Science and Technology, Lanzhou University, Lanzhou 730000, China

**Keywords:** nanocomposites, PVDF, Co_3_O_4_, MWCNTs, DC conductivity

## Abstract

Nanocomposites of polyvinylidene fluoride (PVDF) with dimensional (1D) cobalt oxide (Co_3_O_4_) and *f*-MWCNTs were prepared successfully by the solution casting method. The impact of 1D Co_3_O_4_ filler and 1D Co_3_O_4_/*f*-MWCNTs co-fillers on the structural, thermal, and electrical behavior of PVDF were studied. The crystal structural properties of pure PVDF and its nanocomposite films were studied by XRD, which revealed a significant enhancement of β-phase PVDF in the resulting nanocomposite films. The increase in β-phase was further revealed by the FTIR spectroscopic analysis of the samples. TG, DTA, and DSC analyses confirmed an increase in thermal stability of PVDF with the addition of nano-fillers as well as their increasing wt.%. From impedance spectroscopic studies, it was found that the DC conductivity of PVDF increases insignificantly initially (up to 0.1 wt.% of nano-fillers addition), but a significant improvement in DC conductivity was found at higher concentrations of the nano-fillers. Furthermore, it was observed that the DC conductivity decreases with frequency. The increase in DC conductivity corresponded to the strong interactions of nano-fillers with PVDF polymer chains.

## 1. Introduction

Poly (vinylidene fluoride) (PVDF) is one of the most important polymers and exists in four various crystalline forms, i.e., α, β, δ, and γ phases. Of all the polymorphs, the polar β-phase is the most attractive for researchers because of its technologically important characteristics, such as a high dielectric constant and pyro and piezoelectric properties [1,2]. Therefore, PVDF is used extensively in applied research and is equally preferred in technological applications [3,4,5]. For example, PVDF has found prospective uses in the field of electronics, actuators, sensors, optics, biological imaging, batteries, transducers, electro-optical devices, and membranes [6]. Several methods, such as crystallization from solution, mechanical deformation, high-pressure melt crystallization, and applying a strong electric field, have been utilized to induce the β phase in PVDF [7]. Among them, the solution casting method is preferred owing to the fact that the polar β-phase can be created easily by reinforcing PVDF with a suitable filler, particularly nano-based conducting materials [8].

Nano-fillers are nano-sized structures such as clay, multi-wall carbon nanotubes (MWCNTs), metal oxides, etc., which display a significant role in the conversion of non-polar α-phase PVDF to polar β-phase on the one hand, and on the other, improves its thermal and electrical properties. However, the extent of the induction of these properties into the resulting PVDF nanocomposites is limited by the poor dispersion of nano-fillers in the matrix [9]. For example, it has been observed and reported that using pristine MWCNTs as reinforcement impacted the PVDF conversion to β-phase insignificantly. This was owed to the weak interfacial interaction of MWCNTs with the polymer chains. However, a significant improvement in β-phase formation was observed by using functionalized MWCNTs (*f*-MWCNTs) as nano-fillers [7]. The enhancement in the dispersibility of MWCNTs corresponded to the enhanced interfacial interactions of the MWCNTs with PVDF upon the functionalization of the former [10]. Although functionalized MWCNTs have served the purpose to some extent, the high cost of MWCNTs is one of the grave concerns for the potential application of such PVDF nanocomposites. Because of this reason, low-cost alternative fillers such as metal oxides, including TiO_2_, SnO_2,_ ZnO, etc., have received incredible attention during the last couple of years. Moreover, the matrix and contents of these metal-oxide-based nano-fillers have been shown to be more effective in altering the properties of PVDF [11]. However, the structure (geometrical shape) of the nano-filler is important for altering the characteristics of the matrices [12]. One of the examples is MWCNTs, which have a one-dimensional (1D) nano-architecture and show a tremendous impact on the β-phase formation of PVDF. Therefore, in this paper, we focus on the use of 1D metal-oxide nano-fillers for tailoring the properties of the resulting PVDF nanocomposites [13]. It is pertinent to mention here that anisotropic, e.g., one-dimensional (1D) nanomaterials, have played a significant role in developing new devices for practical applications in diverse areas, including but not limited to catalysis, sensors, field effect transistors, and energy storage systems [14,15,16,17,18,19].

Co_3_O_4_ is widely used as an anode material in Lithium-ion batteries (LIBs) owing to its high theoretical-based energy storage capacity of 890 mAhg^−1^. Therefore, various preparation processes have been utilized to synthesize nanoscale structures of Co_3_O_4_ with different morphologies and have been used for different purposes, including as nano-fillers in a number of polymeric materials [20,21]. However, to the best of our knowledge, it has not been used as a 1D nano-filler alone or in combination with *f*-MWCNTs for the formation of PVDF composites.

Therefore, here in this article, we present the preparation of PVDF nanocomposites with 1D nanostructures and explore their impact on the different properties of the resulting PVDF nanocomposites. Properties, such as thermal stability and electrical conductivity, were thoroughly investigated as a function of the nature and concentration of nano-fillers in the resulting nanocomposites.

## 2. Experimental Section

### 2.1. Materials

Powder Poly (vinylidenefluoride) (PVDF), (average molecular weight; M_w_~534,000), polyvinylpyrrolidone (PVP), with an average molecular weight; M_w_~1,300,000, tetrahydrofuran (THF), dimethylformamid (DMF), sulphuric acid (H_2_SO_4_), nitric acid (HNO_3_), and finally, cobalt nitrate hexahydrate [Co(NO_3_)_2_·6H_2_O] were acquired from Sigma-Aldrich. Multi-wall carbon nanotubes (MWCNTs) were used after functionalization in our own research lab following the reported protocol by Saddiqa Begum et al. [8].

### 2.2. Synthesis of 1D Co_3_O_4_ Nanostructures

The 1D Co_3_O_4_ nanostructures were synthesized by the electrospinning technique. Prior to the electrospinning, the salt solution of the cobalt was prepared by dissolving 1.5 g of Co(NO_3_)_2_·6H_2_O in 10 mL of DMF. After the salt was completely dissolved under constant stirring in DMF, 1.2 g of PVP granules were added, and this mixture was stirred for about 5 h; finally, a clear solution was obtained. A 5 mL syringe was filled with this solution for electrospinning, which was then mounted on the syringe pump. The needle of the syringe was connected to a high-voltage supply while the collector was grounded. The voltage was increased gradually, and the formation of the nanowires started by coming out of the needle, and they were deposited on the metal plate covered with aluminum foil. The distance between the needle tip and the collector surface was set at 15 cm, and a maximum voltage of 15 kV was applied. The nanowires deposited on the collector were then scratched with a spatula and stored in a glass vial. These polymeric cobalt-containing nanowires were then calcined at a temperature of 600 °C to remove the PVP, and finally, the pure Co_3_O_4_ nanostructures were obtained.

### 2.3. Functionalization of the MWCNTs

The functionalized MWCNTs were prepared following the protocol/method published elsewhere [8].

### 2.4. Preparation of Co_3_O_4_/PVDF and Co_3_O_4_/f-CNTs/PVDF Nanocomposites

To prepare the films of the Co_3_O_4_/PVDF, two separate dispersions of the Co_3_O_4_ nanostructures and PVDF were prepared. The Co_3_O_4_ dispersion was prepared by sonicating its powder in a given amount of DMF. Similarly, in a separate centrifuge tube, the proper amount of PVDF was dissolved in DMF and was sonicated at room temperature for about 1 h. Both of these dispersions were mixed together and were kept in the sonicator for further sonication to achieve a homogenous mixture of the two dispersions. This composite dispersion was further refluxed at 70 °C for 6 h followed by 3-h sonication to achieve a satisfactory distribution of the Co_3_O_4_ nano-fillers in the PVDF matrix. Finally, the nanocomposite dispersion was poured into a petri dish and kept in an oven for 5 h at 70 °C. After the given time and temperature, the solvent was completely evaporated, and the Co_3_O_4_/PVDF nanocomposite film/membrane was ready, which was stored for further analysis and applications.

To prepare the Co_3_O_4_/*f*-MWCNTs/PVDF nanocomposite films/membranes, the same procedure was followed with the introduction of *f*-MWCNTS as co-fillers. First, the dispersion of Co_3_O_4_/*f*-MWCNTs was prepared by sonication for 2 h. Meanwhile, the dispersion of the PVDF was prepared separately by sonication. Both of these dispersions were then mixed together, followed by excessive sonication for an appropriate time. The dispersion slurry of these mixtures was then poured into a flat dish and kept in an oven for 6 h, during which the solvent was evaporated, and the composite films of the Co_3_O_4_/*f*-MWCNTs/PVDF were obtained as final products. The *f*-MWCNTs were mixed in three different weight percentages (wt.%), i.e., 0.1 wt.%, 0.15 wt.%, and 0.3 wt.%, while keeping the amount of Co_3_O_4_ and PVDF fixed. In total, five nanocomposite films were prepared, i.e., a blank film that consisted of only PVDF (blank), a Co_3_O_4_/PVDF (PC_1_), and Co_3_O_4_/*f*-MWCNTs/PVDF in which the *f*-MWCNTs amount varied from 0.1 wt.% (PC_1_CNT_1_) to 0.15 wt.% (PC_1_CNT_1.5_) and 0.3 wt.% (PC_1_CNT_3_). A detailed description of the composition of different PVDF nanocomposites films is given in Table 1.

### 2.5. Characterization of the Co_3_O_4_ and Its Nanocomposites

After electrospinning and calcination, the nanostructures of the Co_3_O_4_ were characterized by X-ray diffraction (XRD) analysis, Fourier-transformed infrared (FTIR) spectroscopy, and transmission electron microscopy (TEM). While the nanocomposite films, PVDF, Co_3_O_4_/PVDF, and Co_3_O_4_-*f*-MWCNTs/PVDF, were characterized by XRD, FTIR, thermogravimetric analysis (TGA), thermal differential analysis (TDA), differential scanning calorimetry (DSC), and DC conductivity. The XRD patterns of the samples were taken by Xpert Pro. The diffractometer was equipped with a copper (Cu)-based X-ray source, which yields K-α radiation (λ: 1.542 Å). The FTIR spectra of each of the samples were obtained using a Varian 2000 FTIR-spectrometer attenuated total reflection (ATR) mode in the range of 4000 cm^−1^ to 400 cm^−1^. The JEOL, Japan (Model: JSM 6490) TEM analyzer was used to study the effect of the surface 2D morphology of Co_3_O_4_. The TGA patterns were taken by using a TGA analyzer (Perkin-Elmer TGA-7) in the temperature range ~20–800 °C, at a heating rate of 5 °C min^−1^ under an N_2_ environment. The TDA data were extracted from the TGA patterns. DSC thermograms were taken under a dry N_2_ environment for the nanocomposite films in the temperature range of 0 °C to 200 °C at a heating rate of 10 °C min^−1^. To measure the dielectric properties of the nanocomposite films, an impedance spectrometer was carried out using inductance, L-capacitance, C-Resistance, and R (LCR) meters. The spectra were obtained for the silver (Ag) painted films, which were cut into 10 mm × 10 mm size. The Ag paste was applied to both sides of films for better contact. The silver-painted films were prepared by adding silver metal to isoamyl acetate, which was then mixed well using a brush. The same was coated on blank PVDF and its nanocomposite films. The isoamy acetate was evaporated at a high temperature, in a range from 250 to 450 °C over a few minutes. The silver paste was then used as an electrode to determine the DC conductivity of the thin nanocomposite films [22]. For the impedance measurement, the two red clips, i.e., HD/HS terminals of the LCR meter, were connected to one silver paste side of the film, and the two black clips, i.e., the LS/LD terminals, were connected to the other silver paste side of the film, and the program was run for further processes. The digital LCR meter uses four terminals to apply a current to the test films, with one pair of terminals (High Drive/Low Drive) to measure the impedance across the films with the other pair (High Sense/Low Sense) of terminals.

## 3. Results and Discussion

### 3.1. Analysis of Co_3_O_4_ Nanostructures

**Figure 1:**
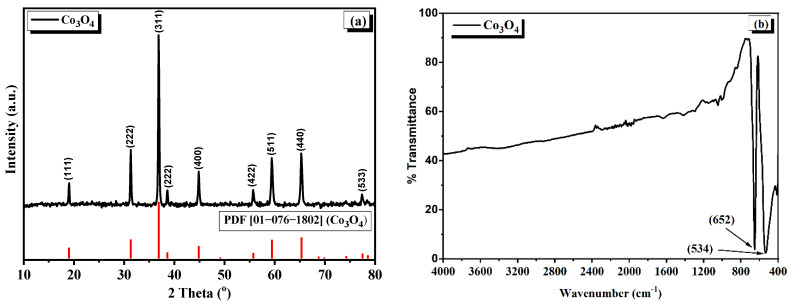
Powder XRD pattern (**a**) and FTIR spectrum (**b**) of Co_3_O_4_ structures prepared by calcination of the as electrospun nanowires.

The XRD pattern of Co_3_O_4_ is given in Figure 1a, which was evaluated for phase identification and the nature of the crystallinity. The diffraction pattern of the Co_3_O_4_ nanostructures correspond to the crystalline phase of Co_3_O_4_ upon matching with the standard (powder diffraction file (PDF) number 01-076-1802), which possesses a cubic spinel crystal system having space group and space group number of Fd3m and 227, respectively. The reflections in the diffraction pattern of Co_3_O_4_ appeared at 2theta positions of 19.02°, 31.31°, 36.90°, 38.60°, 44.88°, 55.74°, 59.45°, 65.34°, and 77.47° which correspond to their respective miller indices (111), (222), (311), (222), (400), (422), (511), (411), and (533), respectively [23]. It is clear from the XRD pattern that there are no impurities present in the Co_3_O_4_ nanostructures, as the peaks that appeared are only in the cubic spinel phase of Co_3_O_4_. Furthermore, the broad hump-like peak usually appears for polymeric materials; in this case, PVF in the XRD patterns is also absent, showing that the electrospun-polymer was removed completely at the selected calcination temperature. The crystallite size was calculated by using Scherrer’s formula, Equation (1).

(1) $$L_{c}=\frac{0.9\lambda }{\beta cos2\theta }$$

The mean crystallite size of Co_3_O_4_ nanostructures was calculated by using Equation (1) and found to be 18.15 nm.

The FTIR spectrum of the crystalline Co_3_O_4_ nanostructures is given in Figure 1b. In addition to the XRD studies, the FTIR study also confirms the formation of spinel-type Co_3_O_4_ nanowires. In the given FTIR spectrum, there are two prominent absorption bands appearing at 534 cm^−1^ and 652 cm^−1^. The absorption band appearing at 534 cm^−1^ is assigned to the Co-O vibration of the B-O atoms (where B is Co^3+^) setting in the octahedral sites of the cubic spinel lattice, and the second absorption band at 652 cm^−1^ is assigned to the vibration of the Co-O of A-O atoms (where A is Co^2+^) setting in the tetrahedral sites of the spinel lattice [1]. No other peaks could be seen in the FTIR spectrum, indicating that the PVP polymer was completely removed at the selected temperature regime.

**Figure 2:**
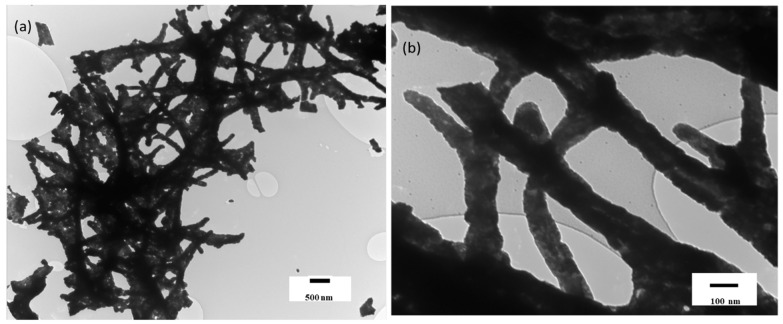
TEM images of Cobalt oxide nanowires at different magnifications, (**a**) low, and (**b**) high.

The morphology of the Co_3_O_4_ nanostructures was studied by TEM, which confirms that the synthesized material is obtained in the form of ID nanostructures. The low- and high-magnification TEM images can be seen in Figure 2a,b, respectively. The surface of these 1D Co_3_O_4_ nanostructures looks compact, grainy, and rough at the edges, while some of the nanostructures have collapsed, which normally happens during the process of calcination. The roughness in the nanowires normally comes due to the elimination of the solvent molecules and PVP, after which the grains fill the space, and the surface becomes rough. The lengths of these nanowires are more than a micrometer (µm), while their diameters range between 104 nm and 194 nm. These 1D nanostructures of Co_3_O_4_ are interwoven, forming a matt-like structure with an enhanced surface area, which will be beneficial in strengthening the PDVF films when mixed with it, providing a better surface interaction.

**Figure 3:**
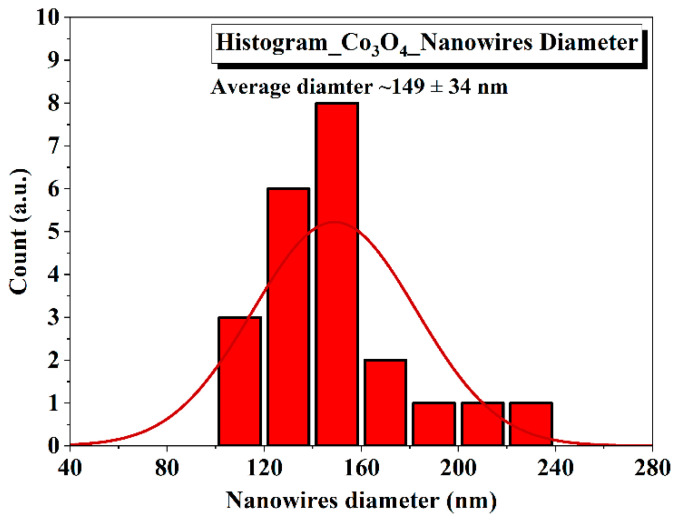
Histogram showing average diameter of Co_3_O_4_ nanostructures.

A histogram diagram which is presented in Figure 3 was plotted to present the average diameter (nm) of Co_3_O_4_ nanostructures. The average diameter calculated from the histogram was 149 ± 34 nm (Figure 3).

### 3.2. Structural Analysis of PVDF Nanocomposite Films

**Figure 4:**
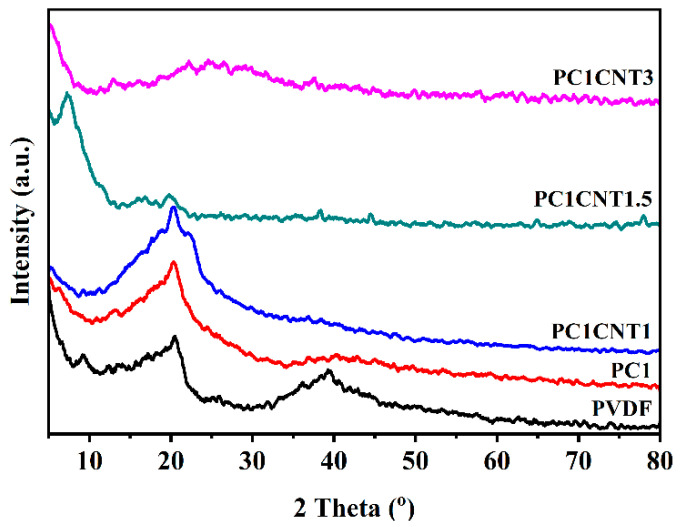
XRD patterns of pure PVDF and PVDF nanocomposites with mono- and bi-nano-fillers prepared by solution cast method, PC_1_, PC_1_CNT_1_, PC_1_CNT_1.5_, and PC_1_CNT_3_.

The XRD analysis was performed over the nanocomposites to see the effect of the filler and co-fillers, i.e., Co_3_O_4_ nanostructures and Co_3_O_4_/*f*-MWCNTs over the crystallinity of the PVDF nanocomposites. Figure 4 shows the XRD patterns of pure (blank) PVDF, Co_3_O_4_/PVDF, and Co_3_O_4_-*f*-MWCNTs/PVDF films in the range of 2theta 5° to 80°. The diffractogram of pure PVDF shows two diffraction peaks at 20.5° and 39.3°, respectively. The peak at 2θ of 20.5° refers to the typical α-phase PVDF, while the peak at 39.3° refers to the (γ) phase PVDF [23,24,25,26]. In the XRD pattern of the Co_3_O_4_/PVDF nanocomposites, one can see that, on the one hand, the diffraction peak at 20.5° has become less intense, and on the other, the peak at 39.43° has nearly vanished. Furthermore, the β-phase peak intensified and showed higher intensity than that which appeared in pure PVDF. This peak appearing at 2theta shows that upon the addition of Co_3_O_4_ nanostructures, a significant transformation from α- to β-phase occurred. Furthermore, the technologically less-important γ-phase no longer exists.

The XRD pattern of the PC_1_CNT_1_ membrane shows one distinct peak at the 2θ position of 20.2° with little deviation from pure PVDF showing reflection for (110). In the XRD pattern of PC_1_CNT_1.5_, the intensity of the main peak of PVDF reduces greatly, and two new peaks develop at the 2θ position of 7.60 and 16.45. The peak at 7.60 is of high intensity, and the peak at 16.96 is comparatively of low intensity. These new peaks appearing as a consequence of the reinforcement of the composite with nano-fillers correspond to the formation of β-phase PVDF. Similarly, the intensity of peaks for α-phase PVDF decreases upon increasing the concentration of the nano-fillers in the resulting composite films (Figure 4). Furthermore, in the case of PC_1_CNT_3_, the XRD pattern gives three low intensities peaks at 2θ positions of 13.23°, 22.27°, and 37.40°. The peak showing the reflection plane (110/200) of the polar β-phase PVDF could be seen in the XRD patterns of all the PVDF nanocomposite-loaded fillers, and the intensity of this peak increased with the increasing number of nano-fillers. From the XRD data, it is obvious that the transformation of α- to β-phase PVDF occurs upon loading of nano-fillers into the nanocomposite on the one hand, and on the other hand, the crystallinity of the resulting films improves.

### 3.3. FTIR Analysis

**Figure 5:**
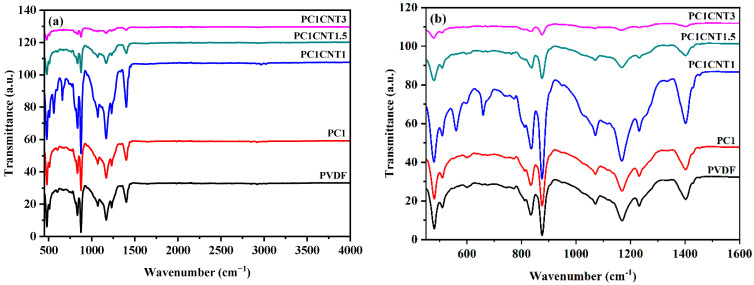
FTIR spectra of pure PVDF and its nanocomposites in the range of 4000 cm^−1^–450 cm^−1^ (**a**), and 1600 cm^−1^–450 cm^−1^ (**b**).

FTIR analysis was carried out to see the effect of the nano-fillers incorporation into the crystal structure and crystallinity of the PVDF films. The FTIR spectra of the blank PVDF film and various wt.% nano-fillers loaded PVDF nanocomposite films are given in Figure 5. The pure PVDF FTIR spectrum shows various absorption peaks at a wavenumber of 479 cm^−1^, 509 cm^−1^, 600 cm^−1^, 876 cm^−1^, 1166 cm^−1^, and 1400 cm^−1^, which corresponds to those reported in the literature [27,28,29]. However, upon the addition of nano-fillers, the peaks for the α- and γ-phase weakened or diminished, and new bands at 559 cm^−1^, 659 cm^−1^, and 772 cm^−1^ appeared. These new peaks correspond to the β- phase PVDF and are thus an indication of the formation of the β- phase [30,31]. The intensities of the β- phase PVDF peaks increase with the increasing nano-fillers content in the resulting nanocomposites. The FTIR results are in good agreement with the XRD data and show that the crystallinity of PVDF was enhanced with the addition of nano-fillers. Furthermore, the amount of β-phase in the resulting PVDF nanocomposites was enhanced.

### 3.4. Thermal Analysis of PVDF Nanocomposite Films

**Figure 6:**
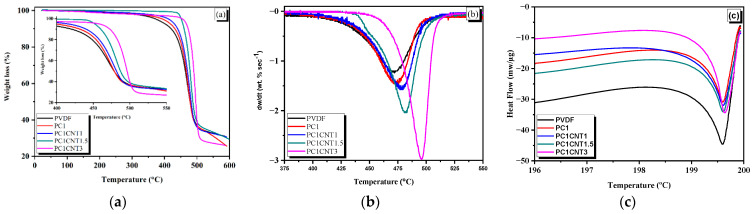
TG (**a**), DTA (**b**), and DSC (**c**) curves of blank and different weight percent loaded nano-fillers PVDF nanocomposites films.

The prepared nanocomposite films of PVDF with single (Co_3_O_4_) and co-nano-fillers (Co_3_O_4_/*f*-MWCNTs) were subjected to thermal analysis in order to see the impact of these nano-fillers upon the thermal behavior of the prepared films. Figure 6a shows the TGA curves of blank PVDF film and its nanocomposites. The films were heated continuously from 25 °C to 600 °C with a heating rate of 10 °C min^−1^. As shown in Figure 6a, the blank film of the PVDF is stable up to 340 °C, after which it follows a one-step degradation. The total weight loss calculated is approximately 66%. The residue may be carbonaceous material as the degradation was carried out under an N_2_ atmosphere. The nanocomposite films of PVDF under study are also showing a single-step thermal degradation process similar to that of the pure PVDF. However, there is a significant difference in the thermogram of pure PVDF and those of nanocomposite films in terms of their thermal degradation temperature and the remaining residue. The TGA pattern of PC_1_ (Co_3_O_4_/PVDF nanocomposite) shows an onset temperature (T_onset_ = 374 °C) higher than that of pure PVDF while lesser weight loss (~60%).

The thermal stability of PVDF increases upon the formation of its nanocomposites with the nano-fillers. An increase in the thermal stability of the polymers upon reinforcing with fillers was also observed previously [32,33,34,35]. Furthermore, the thermal stability increases with increasing the amount of the nano-fillers in the resulting nanocomposites. As can be seen in Figure 6a, T_onset_ was enhanced to 401 °C, 440 °C, and 449 °C upon PVDF nanocomposite formation with Co_3_O_4_/(0.1 wt.%)*f*-MWCNTs, Co_3_O_4_/(0.15 wt.%)*f*-MWCNTs and Co_3_O_4_/(0.3 wt.%)*f*-MWCNTs, respectively. The shift in T_onset_ corresponds to the better interaction of the polymer with the nano-fillers. Mendes et al. [2] explained that the enhancement in thermal degradation temperature was as a result of the increased wt.% of silica on the basis that silica nanoparticles do not allow the volatility of the decomposed product during pyrolysis, hence restricting the continuous decomposition of PVDF. The other reason for this high stability against thermal degradation may be due to the large number of ceramic particles that provide effective thermal shielding. Here in our case, the strong interaction between the nano-fillers and the polymer chains enabled the resulting nanocomposites to withstand heating.

The differential thermal analysis (DTA) data of the pure PVDF film and its nanocomposites (Co_3_O_4_/PVDF and Co_3_O_4_/*f*-MWCNTs/PVDF) are presented in Figure 6b. The peak temperatures (T_p_) of all the samples were analyzed, and it was observed that the T_p_ of PVDF increases with the addition of nano-fillers as well as the concentration of nano-fillers. The values of different temperatures are given in Table 2.

Furthermore, the thermal behavior of pure PVDF and its nanocomposites with Co_3_O_4_ (PC_1_) and Co_3_O_4_/*f*-MWCNTs (0.1 wt.% PC_1_CNT_1_, 0.15 wt.% PC_1_CNT_2_, and 0.3 wt.% PC_1_CNT_3_) were carried out by DSC at a heating rate of 20 °C/min. The DSC thermograms are presented in Figure 6c. The aim of the DSC analysis was to study in detail the phase change of PVDF upon the loading of nano-fillers [36]. The DSC study of the Co_3_O_4_/PVDF and Co_3_O_4_/*f*-MWCNTs nanocomposites shows that the melting temperature (T_m_) of PVDF increases upon the addition of nano-fillers and their concentration compared to that of pure PVDF film. Although the increase in melting temperature is small, a trend is clearly shown. This increase in T_m_ as a function of the addition of nano-fillers and their concentration is corroborated by the phase change of PVDF from α to β, which is further owed to the better distribution of reinforcement in the polymer matrix. [37,38]. The pertinent values of the T_m_ are given in Table 2.

### 3.5. Electrical Behavior of PVDF Nanocomposites Films

**Figure 7:**
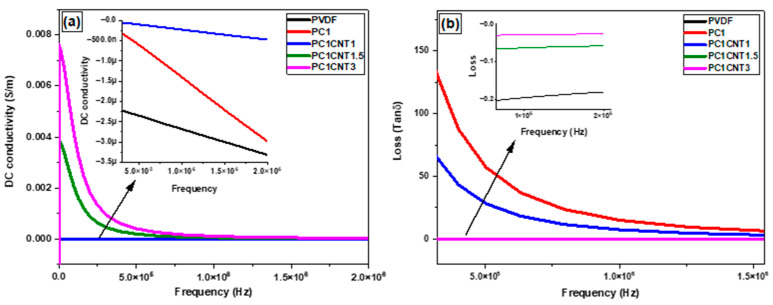
DC Conductivity (**a**), and dielectric loss (**b**) of PVDF and its composites with mono- and bi-nano-fillers consisting of different wt.% of MWCNTs.

The prepared PVDF films with an incorporated amount of Co_3_O_4_ and Co_3_O_4_/*f*-MWCNTs nano-fillers were studied for their electrical properties, and the pertinent data are presented in Figure 7a,b. The DC-conductivity of PVDF in the resulting Co_3_O_4_/PVDF and Co_3_O_4_/*f*-MWCNTs/PVDF nanocomposite films were measured in the frequency range of 2.5 × 10^1^ Hz to 2.00 × 10^6^ Hz at room temperature. Pure PVDF has shown very-low conductivity, i.e., 1 × 10^−6^ (S/m), which shows a decreasing trend as the frequency enhances (Figure 7a). Similarly, DC conductivity is also negligible in the case of PC_1_ (Co_3_O_4_/PVDF) and PC_1_CNT_1_ (Co_3_O_4_/(0.1 wt.%)*f*-MWCNTs/PVDF). However, a very good DC conductivity is shown by the PVDF nanocomposites with nanofillers containing 0.15 wt.% (sample PC_1_CNT_1.5_) and 0.3 wt.% (sample PC_1_CNT_3_) *f*-MWCNTs (Figure 7a); as was the case with pure PVD, PC_1_, and PC_1_CNT_1_, the DC-conductivities of samples PC_1_CNT_2_ and PC_1_CNT_3_ also decreased with increasing frequency, initially very quickly, and then slowly. The higher values of DC-conductivity in the cases of PC_1_CNT_1.5_ and PC_1_CNT_3_ could correspond to the increase in polar, i.e., the β-phase of PVDF in the resulting nanocomposites.

The dielectric loss (tan δ) is a critical quantity for the fabrication of electrical storage devices. In this regard, the dielectric losses of the resulting Co_3_O_4_/PVDF and PVDF/Co_3_O_4_/*f*-MWCNTs nanocomposite films were measured at room temperature and plotted as a function of frequency (Figure 7b). As shown in Figure 7b, the tan δ of pure PVDF as well as of the nanocomposites (Co_3_O_4_/PVDF and Co_3_O_4_/*f*-MWCNTs/PVDF) films decreases with increasing frequency. No strong fluctuation in tan δ was observed. This indicates the strong interfacial polarization along the surface of PVDF polymer, whichs follow the Maxwell–Wagner–Sillars (MWS) polarization effect [39,40].

## 4. Conclusions

The pure form of PVDF polymer mostly exists in the α-phase. However, the phase of the polymer can be modified by reinforcing it with suitable nano-fillers. We present here the successful transformation of PVDF from the pre-dominant α-phase to achieve PVDF nanocomposites with a dominant β-phase. To benefit from their anisotropic properties, we have employed 1D nanostructures as nano-fillers. The 1D Co_3_O_4_ nanostructures were prepared by using an electrospinning technique, while the MWCNTs were functionalized to achieve a better dispersion in the PVDF matrix. The synthesized Co_3_O_4_/PVDF and Co_3_O_4_/*f*-MWCNTs/PVDF nanocomposites were characterized by different techniques. The crystal phase transformation and the improvement in crystallinity were confirmed by XRD. Furthermore, FTIR spectroscopy was also used to confirm the formation of dominant β-phase PVDF in the resulting nanocomposites. The thermal stability of the resulting PVDF nanocomposite films was followed by TGA, DTA, and DSC. It was found that the thermal stability of PVDF is significantly enhanced by reinforcement with mono- and bi-nano-fillers. The electrical properties were studied using impedance spectroscopy, and it was found that the DC-conductivity of the nanocomposites increased with increasing nano-fillers content. The enhancement of DC conductivity and the decrease in tan δ with the addition of nano-fillers to PVDF corresponded to the even dispersion of the fillers and co-fillers in the polymer matrix, owing to the strong interfacial interactions of nano-fillers and the polymer. Owing to the enhanced thermal and electrical properties, the fabricated nanocomposites could be used in electronics such as sensors and capacitors.

## Figures and Tables

**Table 1:** Details of Co_3_O_4_ and MWCNTs incorporation of different wt.% in PVDF matrix to prepare the resulting nanocomposite films.

Sample Code	PVDF (wt.%)	Co_3_O_4_ (wt.%)	*f*-MWCNTs
PVDF	100	0.0	0.0
PC_1_	99.9	0.1	0.0
PC_1_CNT_1_	99.8	0.1	0.1
PC_1_CNT_1.5_	99.75	0.1	0.15
PC_1_CNT_3_	99.6	0.1	0.3

**Table 2:** TGA, DTA, and DSC temperatures of pure and nano-fillers loaded PVDF nanocomposites.

Temp	PVDF	PC_1_	PC_1_CNT_1_	PC_1_CNT_1.5_	PC_1_CNT_3_
T_onset_ (°C)	340	374	401	440	449
T_end_ (°C)	496	498	503	508	510
T_P_ (°C)	471	473	478	481	495
T_m_ (°C)	199.59	199.6	199.60	199.61	199.64

## Data Availability

Not applicable.
